# Disruption of NREM sleep and sleep-related spatial memory consolidation in mice lacking adult hippocampal neurogenesis

**DOI:** 10.1038/s41598-020-72362-3

**Published:** 2020-10-05

**Authors:** D. Sippel, J. Schwabedal, J. C. Snyder, C. N. Oyanedel, S. N. Bernas, A. Garthe, A. Tröndle, A. Storch, G. Kempermann, M. D. Brandt

**Affiliations:** 1 Institute of Medical Psychology and Behavioral Neurobiology, University of Tübingen, 72076 Tübingen, Germany; 2 Department of Psychiatry and Psychotherapy, University Hospital Tübingen, 72076 Tübingen, Germany; 3 Max Planck Institute for the Physics of Complex Systems, 01187 Dresden, Germany; 4 Department of Neurology, University Hospital, Technische Universität Dresden, 01307 Dresden, Germany; 5 German Center for Neurodegenerative Diseases (DZNE) Dresden, 01307 Dresden, Germany; 6 Center for Regenerative Therapies TU Dresden, 01307 Dresden, Germany; 7 German Center for Neurodegenerative Diseases (DZNE) Rostock, 18147 Rostock, Germany; 8 Department of Neurology, University of Rostock, 18147 Rostock, Germany

**Keywords:** Non-REM sleep, Adult neurogenesis

## Abstract

Cellular plasticity at the structural level and sleep at the behavioural level are both essential for memory formation. The link between the two is not well understood. A functional connection between adult neurogenesis and hippocampus-dependent memory consolidation during NREM sleep has been hypothesized but not experimentally shown. Here, we present evidence that during a three-day learning session in the Morris water maze task a genetic knockout model of adult neurogenesis (Cyclin D2^−/−^) showed changes in sleep macro- and microstructure. Sleep EEG analyses revealed a lower total sleep time and NREM fraction in Cyclin D2^−/−^ mice as well as an impairment of sleep specific neuronal oscillations that are associated with memory consolidation. Better performance in the memory task was associated with specific sleep parameters in wild-type, but not in Cyclin D2^−/−^ mice. In wild-type animals the number of proliferating cells correlated with the amount of NREM sleep. The lack of adult neurogenesis led to changes in sleep architecture and oscillations that represent the dialog between hippocampus and neocortex during sleep. We suggest that adult neurogenesis—as a key event of hippocampal plasticity—might play an important role for sleep-dependent memory consolidation and modulates learning-induced changes of sleep macro- and microstructure.

## Introduction

In this study, we explore a potential link between adult hippocampal neurogenesis, as a key aspect of structural plasticity in the hippocampus and sleep-dependent mechanisms of memory formation. The medical background of this hypothesis lies in the observation that (1) Alzheimer’s disease as well as age-related regional brain atrophy and episodic memory impairment are associated with changes in sleep and circadian rhythm disruptions^1^ and (2) that chronically impaired sleep results in learning and memory deficits^2^. These findings lead to the assumption that structural changes and sleep disruption may be linked to each other and contribute to cognitive decline in aging and neurodegenerative diseases.

Forming new and lasting memories requires encoding of information by creating a representation in the brain and a consolidation process making the particular information resistant to interference from new, upcoming stimuli. Consolidation does not only stabilize new memories, but it also enhances old ones^3^. The formation of new memories is characterized by structural changes within the involved neuronal systems including synaptic potentiation or depression, growth and pruning of neurites, and changes in vasculature. In the adult hippocampus, we find the unusual situation that plasticity also occurs at the level of entire neurons through adult neurogenesis, not just synapses, and neurites^4^.

In recent years the connection between neurogenesis and hippocampal function has been a vivid field of research, resulting in a number of interesting hypotheses^5–8^. There has been growing evidence that new neurons allow for a better spatiotemporal contextualization of information, that they promote *behavioural pattern separation* and that they facilitate the flexible integration of new information into pre-existing contexts^9–11^. We propose that adult-generated neurons are a particular successful means to solve the plasticity-stability dilemma that any learning network faces^12^. Functional relevance of adult-born neurons has also been shown at the electrophysiological level, as suppression of adult neurogenesis influences the induction of long-term potentiation in the dentate gyrus^9^. Recently it has been postulated that adult hippocampal neurogenesis is also specifically important for long-term memory formation, as Cyclin D2^−/−^ mice with attenuated neurogenesis show deficits in memory retention after 24 h, but not during learning acquisition^13^. Thus, adult-born neurons in particular seem to promote memory consolidation.

The memory consolidation period, which is important for long-term memory retention typically takes place in the absence of further practice. The optimal behavioural state for memory consolidation without any sensory interference is sleep^3,14^.

Many aspects of the relation of sleep and memory have been identified (for an overview see Klinzing et al., 2019^15^). In our current understanding, the networks associated with information acquisition and storage are reactivated during sleep and are strengthened by this process. The hippocampus and the neocortex are two key structures involved in the process of memory formation. During post-learning sleep the hippocampal ensembles, active during wakefulness, are reactivated and interact with neocortical structures^16,17^. The consolidation processes are associated with cellular changes in form of synaptic plasticity^18^.

Rapid eye movement (REM) and non-REM (NREM) sleep complementarily modulate different aspects of specific memory systems. Both sleep states seem to coordinate and promote memory processes by increasing plasticity-related events like immediate early gene expression and consecutive synaptogenesis^17^. Very recently Kumar et al. provided evidence that adult neurogenesis has functional relevance for the consolidation of emotional memory during REM sleep^19^. Whether NREM sleep-related memory consolidation also benefits from adult-born hippocampal neurons has not been investigated so far. However, post-learning reactivation of neuronal ensembles is most consistently observed during NREM sleep^15,20^. In humans, hippocampal pathology-associated cognitive decline seen in Alzheimer’s disease or patients with selective bilateral hippocampal damage is accompanied by a reduction of NREM slow wave sleep^21,22^. These observations prompted us to focus on NREM sleep-related electrophysiological events to investigate the role of adult hippocampal neurogenesis for sleep-dependent memory consolidation.

Slow oscillations and sleep spindles occurring during NREM sleep have been shown to be a prerequisite for memory consolidation^23^. Furthermore, NREM sleep-associated hippocampal sharp-wave ripples (SWRs) have been discussed as a means to transfer information from the hippocampus to numerous regions of the neocortex^24^. The functional relationship is supposedly interdependent, as learning also regulates neuronal activity during sleep, e.g. as change in sleep spindle density during NREM sleep^25,26^. Therefore, sleep EEG recordings are potential surrogates for cognitive functions especially those that depend on cortico-hippocampal interaction. Changes in cortical activity are believed to be driven by sleep-specific and plasticity-related changes in hippocampal activity^27,28^.

The close functional connection between sleep and memory consolidation on the one side, as well as various memory functions and adult hippocampal neurogenesis on the other side suggests that changes in hippocampal neurogenesis influence hippocampal-neocortical network activity in NREM sleep. We thus hypothesize that reducing hippocampal neurogenesis affects sleep-related network reactivation after learning that leads to changes of NREM specific neuronal oscillations and impairment of memory consolidation. We tested this hypothesis in a three-day-long memory experiment in a group of wild-type (WT) mice, and mice with genetically suppressed adult neurogenesis (Cyclin D2^−/−^ mice) associated with robust deficits in long-term memory formation^13,29^. To link memory consolidation with sleep, we compared memory-related changes in sleep EEG across the groups before and during the learning period.

## Materials and methods

### Animals

Experiments were performed on Cyclin D2^−/−^ mice^30^ after rederivation by embryo transfer and backcrossing into the C57BL/6J background for more than six generations. Cyclin D2^−/−^ mice have been shown to be almost completely devoid of adult hippocampal neurogenesis^29^ and to perform worse in certain aspects of neurogenesis dependent learning tasks like the Morris water maze (MWM)^31^. For the control group, WT littermates (Cyclin D2^+/+^) were used. All mice were group-housed at the animal facility of the Center for Regenerative Therapies TU Dresden (CRTD), Dresden, Germany. Animal housing and experiments were performed at controlled ambient temperature (20 ± 2 °C) and humidity (55 ± 10%), and a controlled 12 h light/dark cycle with light onset at 6 a.m. Food and water were available ad libitum except during the water maze intervention.

Only male mice were used because of possible changes in sleep in female mice during different cyclic phases. A total of 24 animals were used. During the experiment, animals were 9–12 weeks old. Four animals had to be excluded for the final analysis because of insufficient EEG/EMG quality (2) or because of insufficient swimming performance (floating) in the MWM (2), resulting in 10 animals in the WT and 10 animals in the Cyclin D2^−/−^ group. All applicable local and federal regulations of animal welfare in research were followed. The experiments were approved by the responsible authority, Landesdirektion Dresden, Germany.

### Implantation of EEG and EMG electrodes

The EEG and EMG implantations were performed under anaesthesia with 100 mg/kg ketamine and 10 mg/kg xylazine. 0.05 mg/kg carprofen was used as a painkiller. The mice were chronically implanted with a synchronous recording system of 1 channel EMG and 2 channel EEG electrodes (8,201, Pinnacle Technology Inc., Lawrence, KS) 10 days before the first recording. The mice were placed into a stereotaxic frame (Stoelting, Wood Dale, IL). The heads were shaved and disinfected with alcohol before midline incisions of 1 cm were made in the scalp. The periostea were wiped away with sterile swabs. The mice received prefabricated EEG and EMG head mounts (8,201, Pinnacle Technology, Lawrence, KS). The head mounts were first fixed to the skull with cyanoacrylate. Four pilot holes were placed in the skulls through openings in the head mounts with 23-gauge needles at the following coordinates relative to bregma: AP: + 2 mm, ML: ± 1.5 mm (EEG2 and ground electrode) and AP: − 4 mm, ML: ± 1.5 mm (EEG1 and EEG common electrode). Four stainless steel EEG screws (8,209, Pinnacle) were then inserted through openings in the head mounts and manually driven into the pilot holes. Silver epoxy was applied to ensure electrical connectivity between the electrodes and the head mounts. Two flexible stainless-steel wires were inserted into the neck muscles to measure the EMG signal in each mouse. The implants were then stabilized and affixed to the skulls with dental acrylic glue and the scalps were sutured. After surgery, all mice were placed in individual clean cages, wrapped in paper to keep them warm and heated with a warming lamp until they woke up. The following days mice received carprofen (0.05 mg/kg) as needed and were given 10 recovery days until the start of the experiment.

### Electrophysiological recordings

All EEG and EMG time series were sampled at 400 Hz. Mice were habituated to the recording box and cable for 48 h before the baseline recording. For the recording, animals were kept single housed in the recording boxes. Starting with the baseline recording, the EEG and EMG signals were continuously recorded until the end of the experiment, only interrupted by the MWM task.

### Sleep scoring

All recordings were divided into non-overlapping consecutive epochs of 4 s. One blinded author (DS) scored each epoch manually as sleep stage “NREM”, “REM” or “Wake”. In addition, each epoch was scored according to whether recording artefacts were present (artefact-contaminated epoch) or not (artefact-free epoch). Manual scoring was performed using our own sleep-staging software *edfView v0.1.8* (https://github.com/jusjusjus/edfView/tree/v0.1.8). An automatic sleep scoring platform was built from these annotations^32^. The wake state is characterized by high EMG activity (active locomotion, higher muscle tone even when resting) and a mixed frequency EEG signal (without a clear peak at a certain frequency in the power spectrum). NREM sleep shows lower EMG activity compared to the wake state as well as a power spectrum peak in the delta band (0.5–4 Hz). REM sleep is characterized by very low EMG activity (with only seldom muscle twitches) and a clear power spectrum peak in the theta band (at 7–8 Hz).

For each mouse, the absolute time spent in a specific stage, duration and number of sleep episodes, NREM and REM sleep episodes were calculated per day. Additionally, the EEG pattern during each sleep state was characterized using the power-spectral density. Epochs containing EEG or EMG artefacts were excluded from spectral analyses.

### EEG event detection

To identify slow oscillations (SO), the EEG signal was first filtered between 0.4 and 4.5 Hz using a phase-neutral fourth-order Butterworth filter and segmented according to the negative-to-positive zero crossings. The segments that fulfilled the following criteria were selected as SO events: the segment had to (i) span an interval between 0.4 and 2.0 s, (ii) it had to belong to the top 35% in terms of their highest negative peak amplitude, and of this selection (iii) the top 45% in terms of their highest negative-to-positive peak-to-peak amplitude were selected. The criteria resulted in the detection of SOs with downstate peak amplitudes exceeding − 80 μV and peak-to-peak amplitudes exceeding 120 μV^33^.

Spindle detection during NREM sleep was also based on procedures described previously^33,34^. The EEG signal was filtered between 10.0 and 16.0 Hz using a phase-neutral fourth-order Butterworth filter. Then, the Hilbert transform was calculated for the filtered signal and smoothed with a moving average of 200 ms. A spindle was identified when the absolute value of the transformed signal exceeded 1.5 standard deviations (SD) of the mean signal in the respective channel, during the animal’s NREM epochs, for at least 0.4 s and not more than 2.0 s. Spindle onset was defined by the time when the signal the first time exceeded the 1.5-SD threshold.

MATLAB scripts used for analyses of SO and spindle detection are available at the URL https://github.com/MedPsych/LongTermMemory_Sleep.

Cross-frequency coupling between SOs and spindles was analysed in the EEG during NREM sleep using the method of phase-amplitude coupling described by Tort et al., 2010^35^. Each consecutive signal of artefact-free NREM EEG was extracted from the recordings. Slow and fast components were delineated by filtering within a slow-wave band (0.4–4.5 Hz) and a spindle band (10–20 Hz) using phase-neutral fourth-order Butterworth filters. The Hilbert transform was applied on each component to extract the phase of the slow and the amplitude dynamics of the fast component. After binning and taking the median of fast amplitudes within 12 equidistant bins of the slow phase, the modulation index (MI) was computed as a measure of phase-amplitude coupling. A detailed description of the properties of MI can be found in Tort et al., 2010^35^. In short, the index is normalized between zero and one. It is proportional to the part of the fast amplitude that modulates with the slow phase, and anti-proportional to the phase-independent part. At excessively low slow-frequency amplitudes, the slow phase cannot be determined with certainty which also reduces the measured MI. We computed MIs using the code in the public online repository https://github.com/jusjusjus/phac-python/tree/v0.0.1.

### Morris water maze task

Hippocampus-dependent learning was assessed with the MWM task. The version used has been described in detail by Garthe et al., 2014^31^. In brief, mice were trained in the reference memory version of the task^36^ to locate a hidden escape platform in a circular pool (200 cm in diameter). The task was performed at lights on (6 a.m.). The water was made opaque with non-toxic white pigment and kept at a temperature of 19–20 °C. Each mouse was given 6 trials per day for 3 consecutive days with an intertrial interval of about 30 min. Mice were released from one of three possible starting points and allowed to search for the platform for up to 120 s. Each day the starting position was changed, while it was kept constant during the day. At the end of the trial, irrespective of trial performance mice were guided to the platform and allowed to remain there for 15 s. Swim paths were recorded using Ethovision, Version XT 7 (Noldus, Wageningen, the Netherlands). Further analyses were done based on the raw time-tagged xy-coordinates using MATLAB, Version 2017a (The Mathworks, Ismaning, Germany).

Swimming paths, time to target and time in quadrants were analysed using the Ethovision software. In addition, we used route efficiency as a measure that is very robust to variations in swimming speed of individual mice and therefore differences in trial path length, when mice did not reach the platform. It is defined as:

$$\text{Route Efficiency} [\%] = \frac{1} {\text{actual path length of trial}} * \text{direct path length of trial} * 100$$

The *direct path length of trail* is defined as a straight line from the drop position to the platform.

Search strategies were classified according to parameters and an algorithm described in previous studies^9,31^, originally based on Balschun et al., 2003^37^. Briefly, when repeatedly performing the MWM, mice show a sequential use of specific search strategies. These range from undirected random search in the beginning to spatially precise search strategies that are also more efficient after several trials. We defined seven different search strategies. The first four search strategies (1: thigmotaxis, 2: random search, 3: scanning, and 4: chaining) represent undirected search patterns. Search strategies 5, 6, and 7 (directed search, focal search, direct swimming) are specified by a directional preference for the goal position from different starting points, suggesting a contribution of an internal representation, or an internal spatial map and are thus hippocampus-dependent. Search strategies were quantitatively evaluated using a point system. A trial with undirected search received zero points, directed search 1 point, focal search 2 points, and direct swimming 3 points. This reflects the increased sophistication of the internal map of the MWM in the spatial precision strategies with direct swimming constituting the most advanced search strategy requiring a well-developed spatial map as an internal representation. For a more detailed description of search strategies and their sequential use, see Garthe et al., 2014^31^.

### Tissue preparation and immunohistochemistry

After the EEG recording period, the animals were put into deep anaesthesia with 100 mg/kg ketamine and 10 mg/kg xylazine and perfused transcardially with ice-cold NaCl 0,9%. The brains were removed from the skulls, post-fixed in 4% paraformaldehyde in phosphate-buffered saline (PBS), pH 7.4 overnight, and then transferred into 30% sucrose solution in PBS, pH 7.4. The brains were cut into 40 μm thick coronal sections on a dry-ice-cooled copper block on a sliding microtome (SM 200R; Leica, Bensheim, Germany).

As adult neurogenesis depends on precursor cell divisions in the dentate gyrus (DG), cell proliferation in the subgranular zone can, in this context, be used as proxy measure to indicate the absence of adult neurogenesis in the Cyclin D2^−/−^ mice. To detect cell proliferation Ki67 was used as a marker. Free-floating sections were incubated in 0.6% H_2_O_2_ for 30 min to inhibit endogenous peroxidase activity. After washing, non-specific antibody-binding sites were blocked using 10% donkey serum and 0.2% Triton-X100 in Tris-buffered saline (TBS) for 1 h at room temperature. The primary antibody rabbit anti-Ki67 (Novocastra, 1:1,000) was applied overnight at 4 °C. Sections were incubated with biotinylated secondary antibodies for 2 h at room temperature (1:500, Dianova). Primary and secondary antibodies were diluted in TBS supplemented with 3% donkey serum and 2% Triton-X10. Detection was performed using the Vectastain ABC-Elite reagent (9 μg/ml of each component, Vector Laboratories, LINARIS) with diaminobenzidine (0.075 mg/ml; Sigma) and 0.04% nickel chloride as a chromogen. All washing steps were performed in PBS. Sections were mounted onto glass slides, cleared with Neo-Clear (Millipore) and cover-slipped using Neo-Mount (Millipore). Ki67-positive cells were counted, by applying the simplified version of the optical fractionator principle as previously described^38^ on every sixth section along the entire rostro-caudal axis of the dentate gyrus, using a brightfield microscope (Leica DM 750).

### Statistical analysis

The swimming metrics for each group (n = 10) from days 1, 2, and 3 (6 trials per day) were compared using one way repeated-measure ANOVA with post-hoc Bonferroni-adjusted two-sided t-test as appropriate. Where sphericity was rejected by the Mauchly test, the Greenhouse–Geisser correction was applied. For comparison of learning induced changes in sleep parameters, variables from day 1, 2, and 3 were pooled in the learning condition and compared to those at baseline using paired two-sided t-tests for each group (n = 10). Unpaired two-sided t-tests were performed for single group comparison of Ki67 data and EEG parameters under baseline and learning conditions, respectively. Normality of variables was verified with the Shapiro–Wilk-test. For all correlations a linear regression model was used. Correlations between variables were measured using Pearson’s correlation coefficient with two-sided tests. All statistical tests were computed using SPSS 25.0, and SciPy v1.2.0.

### Experimental design

**Figure 1:**
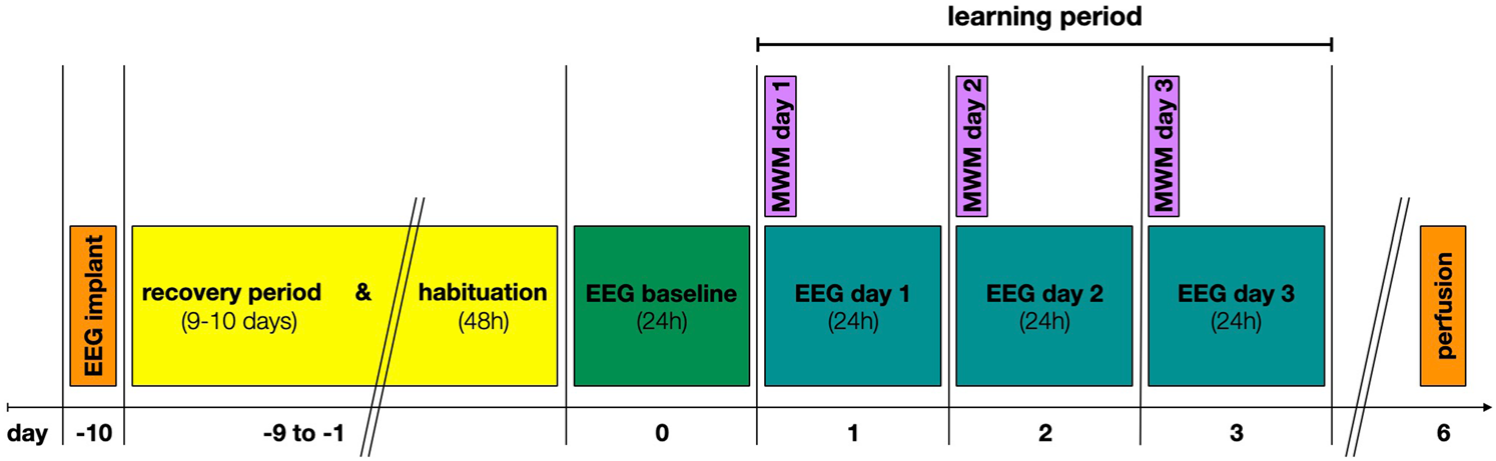
Experimental design. EEG/EMG implantation took place at day-10, mice were then allowed 9–10 days of recovery including a 48 h period of habituation to the recording setup. This was followed by a 24 h baseline EEG/EMG recording. EEG/EMG signals were continuously recorded for the next 3 days (EEG day 1–3). Each morning at 6 a.m. on day 1–3 the Morris water maze (MWM) was performed. These 3 days are summarized as the learning period. Mice were perfused for histological analysis afterwards.

The experimental procedure was the same for both groups, i.e. WT and Cyclin D2^−/−^ mice. After EEG and EMG implantation surgery mice were allowed 10 days of recovery. Right after, animals were habituated to the recording setup for 48 h. On the day after, the EEG and EMG signals were continuously recorded for 96 h (until the following morning after the last MWM day). The EEG and EMG were only briefly disconnected for each trial during the MWM task. Mice were perfused afterward for tissue analysis. The experimental timeline is summarized in Fig. 1. During the experiments, as well as during data analyses, the experimenters were blinded with respect to the animals’ group affiliation.

## Results

### Attenuation of adult hippocampal neurogenesis in Cyclin D2^−/−^ mice

**Figure 2:**
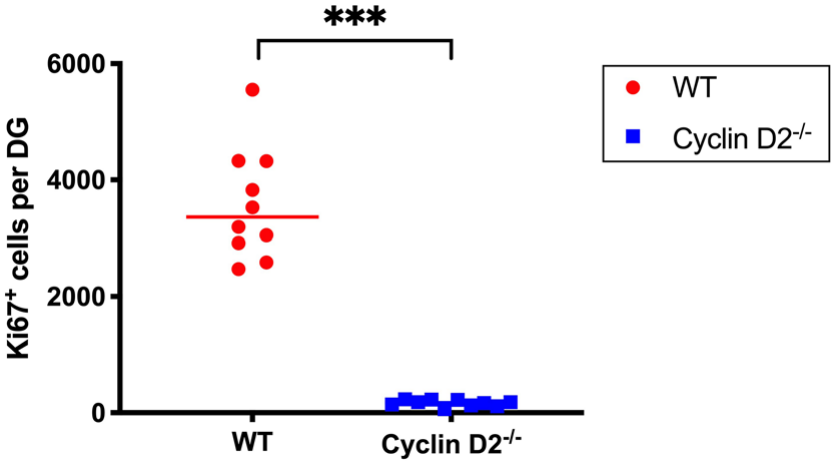
Cell proliferation. WT red, Cyclin D2^−/−^ blue. Ki67^+^ cells per DG as a measure for neurogenesis levels (proliferating cells) in both groups (n = 10 per group; two-sided t-test; ***: p < 0.001).

Cyclin D2^−/−^ mice are known to almost completely lack adult hippocampal neurogenesis^13,29^. In rodents, adult hippocampal neurogenesis is dependent on the mitotic activity of neuronal stem- and precursor cells in the subgranular zone of the DG. The proliferating cells in the DG were identified using Ki67, a protein expressed during the cell cycle. The WT mice revealed 3,577 ± 951 Ki67^+^ cells per DG, indicating a normal level of hippocampal neurogenesis with some inter-individual variance (Fig. 2). In Cyclin D2^−/−^ mice only very few cells were Ki67^+^ (166 ± 56), indicating that the genetic suppression of neurogenesis was equally effective for all animals in the Cyclin D2^−/−^ group (p < 0.001).

### Impaired performance of Cyclin D2^−/−^ mice in the Morris water maze learning task

**Figure 3:**
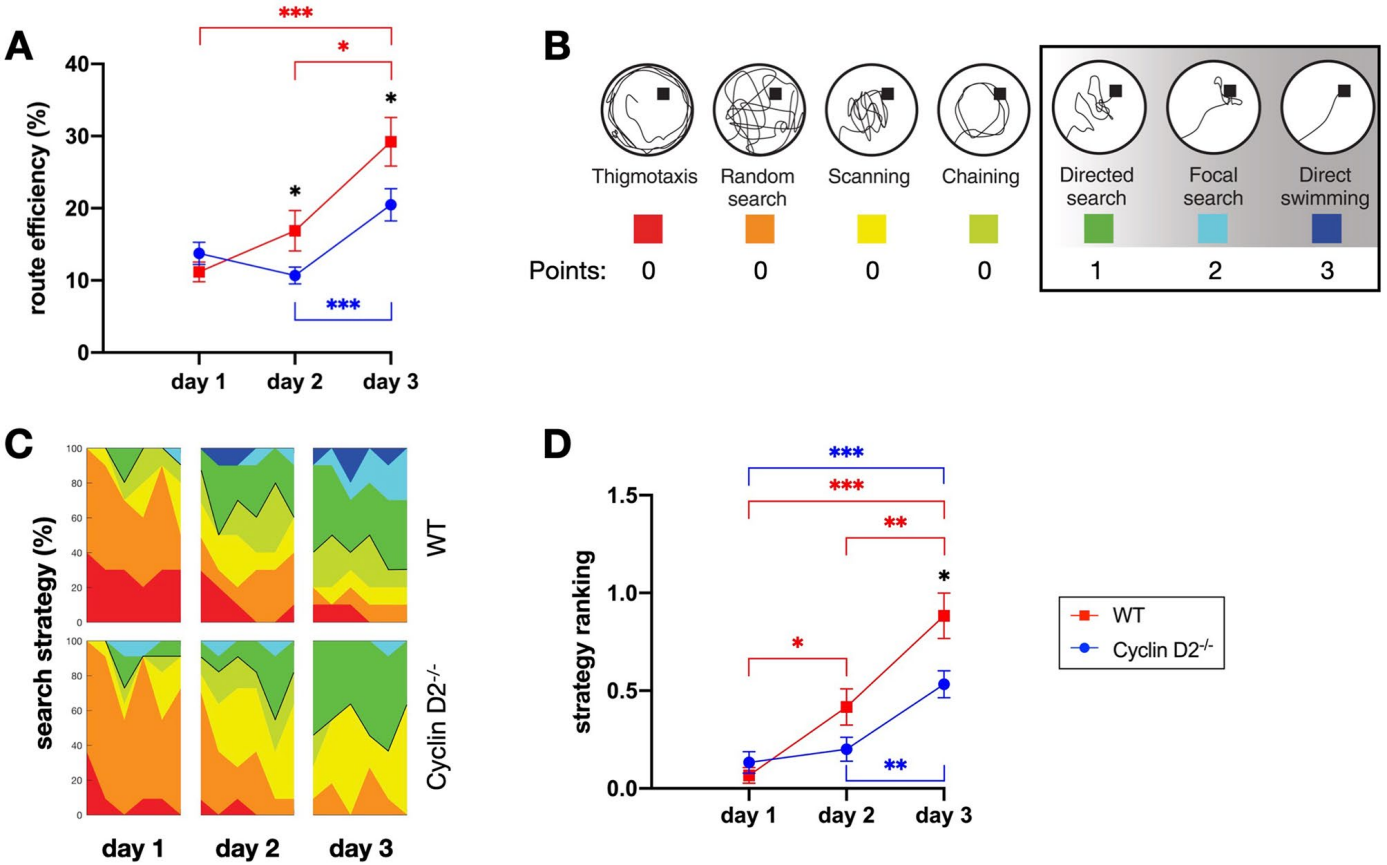
MWM performance. WT red, Cyclin D2^−/−^ blue. (**A**) Mean route efficiency in % (100% = direct swimming path) for each day (6 trials per day) in both groups. (**B**) Discrimination and ranking of different search strategies. Hippocampus-independent strategies were ranked with 0 points and only hippocampus-dependent strategies (direct search, focal search, and direct swimming) were ranked with 1, 2 or 3 points. (**C**) Proportional appearance in % of different search strategies over all trials during the 3 days of learning the MWM task in both groups. (**D**) Mean strategy ranking for each day (6 trials per day) in both groups. (n = 10 for each group, 6 trails per day; one-way repeated-measure ANOVA with post-hoc Bonferroni-adjusted two-sided t-test as appropriate; *: p < 0.05; **: p < 0.01; ***: p < 0.001; data presented as mean ± 1 SEM).

As a quantitative proxy measure for learning success in the MWM we used the outcome *route efficiency*. This measure represents the optimality of the swimming path and is defined as the quotient of the direct path to the platform and the one that the animal actually swam in a given trial. As previously described, mice also use qualitatively different search strategies to locate the hidden platform in the MWM^9,31,39^. In our current experiment, they progressed from undirected to directed search patterns over the course of multiple trials and days. To quantify the progression, each trial was assigned to one of seven strategy categories and scored accordingly (see Fig. 3B). We used a rating system (points) to reflect the growing sophistication of hippocampus-dependent directed search patterns.

On day 1, the route in the water maze was on average eight times longer than optimal with no significant difference between the groups (route efficiency: 11.2 ± 1.4% for WT; 13.8 ± 1.5% for Cyclin D2^−/−^; p = 0.212; Fig. 3A). Mostly undirected search strategies were observed independently of the group on the first day (strategy ranking: 0.7 ± 0.04 for WT; 0.13 ± 0.06 for Cyclin D2^−/−^; p = 0.333; Fig. 3D). On the following days, both groups found the platform more efficiently (repeated-measure ANOVA: p < 0.001 for WT; p = 0.001 for Cyclin D2^−/−^; Fig. 3A), and were using more directed search strategies (repeated-measure ANOVA: p < 0.001 for both groups). Compared with WT mice, routes of Cyclin D2^−/−^ mice were 6.2% less efficient on day 2 (p = 0.045), and 8.7% on day 3 (p = 0.033). WT mice showed a higher strategy ranking on day 2 (p = 0.055) and day 3 (p = 0.011) (Fig. 3C, D). For additional readouts concerning time to target, time spent in target quadrant and swim speed, see Suppl. Figure 1.

The group comparison in the MWM task revealed that the Cyclin D2^−/−^ group, lacking adult-born hippocampal neurons, showed a lower hippocampus-dependent spatial learning performance on days 2 and 3, while the initial performance on day 1 was similar in both groups. Days 2 and 3 of the MWM represented the performance after post-learning sleep, where mice were able to consolidate the newly formed memory from the day(s) before.

### Differences in sleep macrostructure of Cyclin D2^−/−^ mice under baseline and learning conditions

EEG and EMG recordings were performed continuously for 4 days (except during the swimming task). Starting with a baseline measurement of 24 h before the first MWM trial (MWM day 1) and continuing during the 3 consecutive days of learning the MWM until the morning of the 4th day (overall 96 h of EEG and EMG recordings per animal).

**Figure 4:**
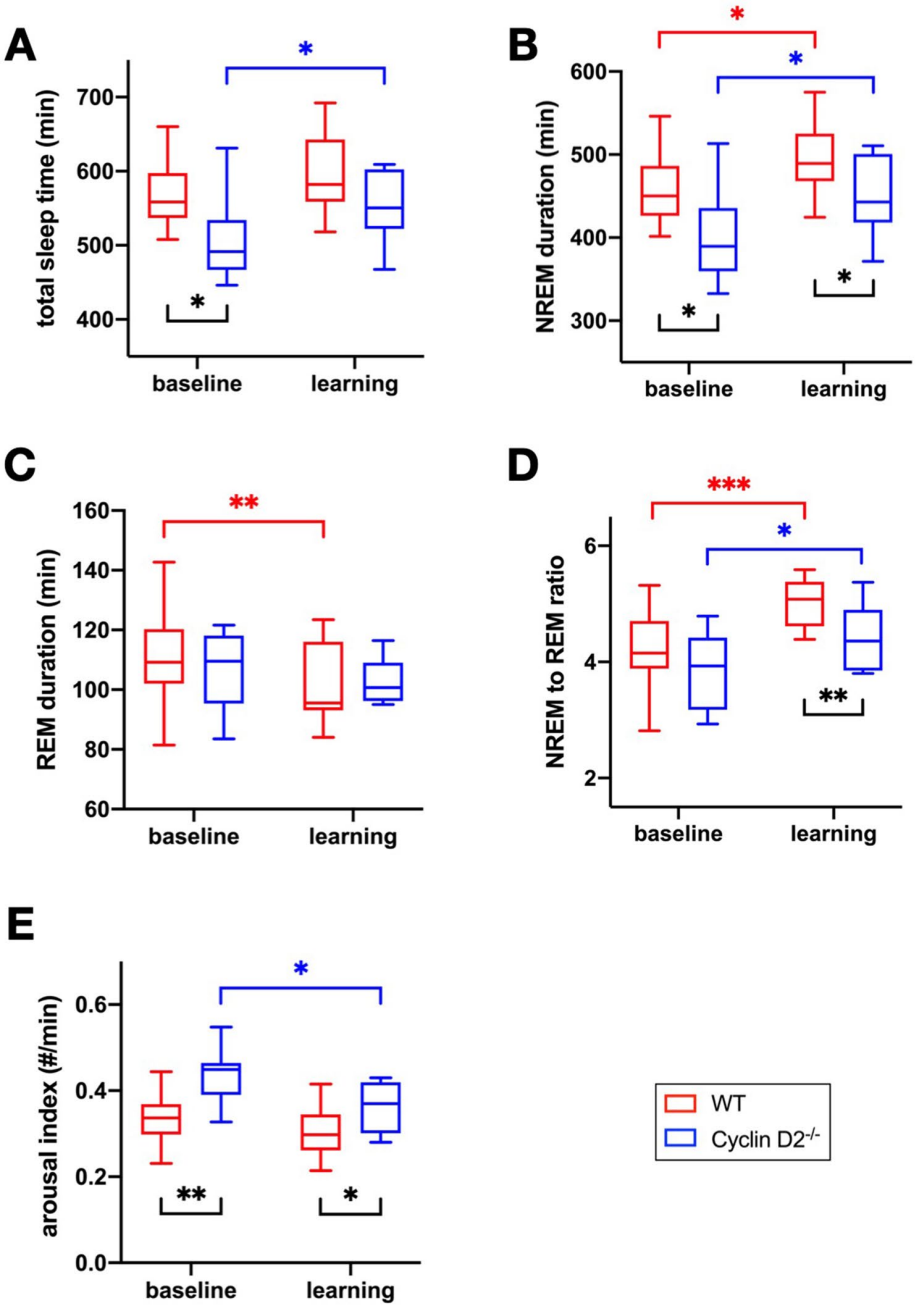
Sleep macrostructure. WT red, Cyclin D2^−/−^ blue. Each panel shows the group comparison per 24 h recording at baseline and during the learning period (mean of days 1–3). (**A**) Total sleep time (TST) in min. (**B**) NREM sleep duration in min. (**C**) REM sleep duration in min. (**D**) NREM to REM sleep ratio. (**E**) Arousal index defined as arousals per minute. (n = 10 per group; two-sided unpaired t-test for group comparison and two-sided paired t-test for baseline learning comparison; *: p < 0.05; **: p < 0.01; ***: p < 0.001).

Prior to the learning stimulus (baseline), Cyclin D2^−/−^ mice showed a significantly shorter total sleep time (TST) of 11% (p = 0.021), and 12% less NREM sleep duration (p = 0.021) compared to WT animals (Fig. 4A,B), while REM sleep duration and NREM to REM sleep ratio was similar in both groups (Fig. 4C,D). Thus, differences in TST were mainly due to discrepancies in NREM sleep duration. The arousal index represents the rate of arousals that occur during total sleep time. It is a marker for sleep fragmentation and therefore inversely related to sleep quality. The arousal index was about 30% higher in Cyclin D2^−/−^ mice (p = 0.001) at baseline, representing a higher sleep fragmentation in Cyclin D2^−/−^ compared to WT animals.

During learning (MWM days 1–3), only Cyclin D2^−/−^ mice showed an increase in the mean TST per 24 h during the learning period (p = 0.034; Fig. 4A), abolishing the group difference of TST at baseline. Both, WT and Cyclin D2^−/−^ mice, exhibit an increase of mean NREM sleep duration per 24 h during the learning days (p = 0.018 for Cyclin D2^−/−^; p = 0.025 for WT; Fig. 4B). The WT group showed a decrease in mean REM sleep duration compared to baseline (p = 0.005), while the mean REM sleep duration in the Cyclin D2^−/−^ group remained unchanged (Fig. 4C). The NREM to REM sleep ratio increased in both groups (p = 0.01 for Cyclin D2^−/−^; p < 0.001 for WT). However, the WT group showed a significantly higher NREM to REM sleep ratio during the learning period as compared to Cyclin D2^−/−^ (p = 0.008; Fig. 4D) which represents more NREM sleep at the expense of REM sleep.

In summary, the Cyclin D2^−/−^ mice showed a reduced TST and NREM duration but higher arousal index at baseline. During learning both, WT and Cyclin D2^−/−^ mice showed a prolonged NREM sleep duration but seemed to use different regulative patterns. While Cyclin D2^−/−^ animals reached more NREM sleep by a prolonged TST, WT mice used a more prominent shift towards a higher NREM sleep proportion while keeping TST at a stable level.

Cyclin D2^−/–^ induced differences of the arousal index abated from 30% at baseline to 19% during the learning period (p = 0.042).

### Differences in sleep microstructure in Cyclin D2^−/−^ mice under baseline and learning conditions

SOs and sleep spindles occurring during NREM sleep have been identified as important electrophysiological markers for memory consolidation during sleep^3^. The density of sleep spindles and the coupling with SOs increase after hippocampus-dependent memory tasks^25,26,40,41^. The timing of spindles within the SO cycle is hypothesized to ensure proper integration of cortical and subcortical systems during the formation of memories, a process that has been described as phase-amplitude coupling (PAC)^41–43^. Beside the memory promoting function sleep spindles consolidate NREM sleep and elevate arousal threshold^44^.

**Figure 5:**
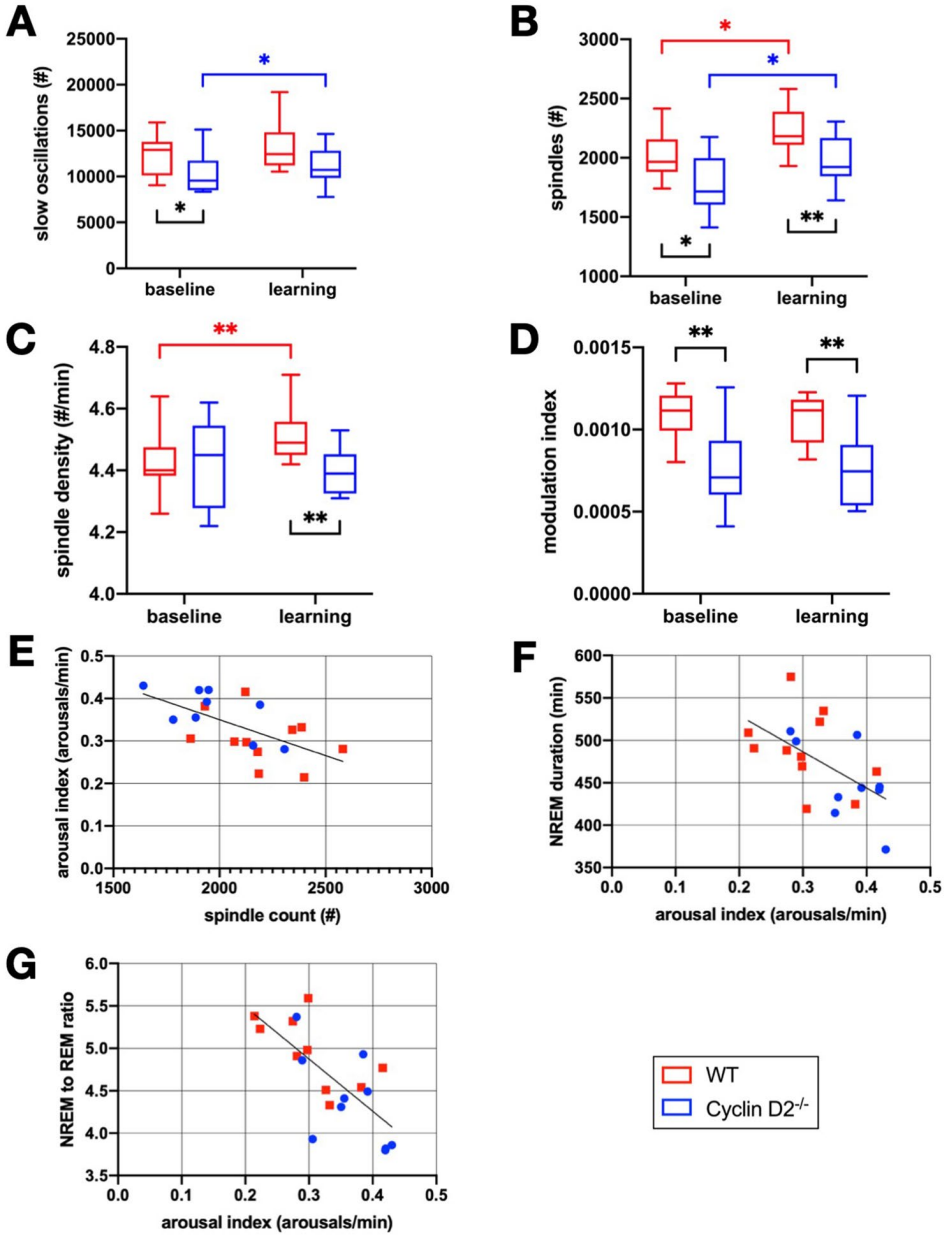
Sleep microstructure and arousal index correlations. WT red, Cyclin D2^−/−^ blue. Each sub-figure shows the group comparison per 24 h recording at baseline and during the learning period (mean of days 1–3). (**A**) Slow oscillation count. (**B**) Sleep spindle count. (**C**) Sleep spindle density in count per min. (**D**) Modulation index of phase-amplitude coupling (PAC). (**E**) Correlation of sleep spindle count with arousal index (r = − 0.612; p = 0.004). (**F**) Correlation of arousal index NREM sleep duration (r = − 0.573; p = 0.008). (**G**) Correlation of arousal index with NREM to REM sleep ratio (r = − 0.722; p < 0.001). (n = 10 per group; two-sided unpaired t-test for group comparison and two-sided paired t-test for baseline learning comparison; *: p < 0.05; **: p < 0.01; Pearson’s correlation coefficient with two-sided test).

At baseline, Cyclin D2^−/−^ mice showed a reduced count of SOs (p = 0.041) and spindles (p = 0.023) compared to WT animals (Fig. 5A, B). This was most likely due to a shorter NREM sleep duration as the SO and spindle density was similar in both groups (Fig. 5C, Suppl. Figure 1). PAC analysis revealed a reduction of the modulation index in Cyclin D2^−/−^ mice (p = 0.001; Fig. 5D). Reduced dependence of spindle amplitude on SO cycle could be interpreted as an impairment in cortical-subcortical coordination in Cyclin D2^−/−^ mice.

During the learning period, the SO count increased in the Cyclin D2^−/−^ group (p = 0.029), while SO density remained unchanged (see Suppl. Figure 1). There was no difference in SO count in the WT group compared to baseline (Fig. 5A). Spindle count increased in both groups (p = 0.01 for WT; p = 0.018 for Cyclin D2^−/−^), but remained higher in WT animals during the learning period (p = 0.007; Fig. 5B). Spindle density, however, increased progressively over the course of the learning period only in the WT group (Fig. 5C, Suppl. Figure 1). This resulted in a lower spindle density during the learning days for Cyclin D2^−/−^ mice compared to WT animals (p = 0.004; Fig. 5C). There were no learning-induced changes of PAC in both groups (Fig. 5D). For single day sleep data please refer to supplementary information (Suppl. Figure 2).

Quantitative analyses of memory-related cortical activity patterns during sleep (SOs and spindles) reflected differences and changes of NREM sleep duration. However, spindle density as a parameter of NREM sleep quality showed a divergent change in the two groups during the learning period.

The mean count of spindles per 24 h of sleep inversely correlated with the arousal index (r = − 0.612; p = 0.004; Fig. 5E). A lower arousal index was associated with an increased NREM sleep duration (r = − 0.573; p = 0.008; Fig. 5F) and NREM to REM sleep ratio (r = − 0.722; p < 0.001; Fig. 5G). These findings are in line with the hypothesis that spindles elevate the arousal threshold leading to a stabilization of NREM sleep^44^.

### Correlation of adult neurogenesis rate with NREM sleep in WT mice

The previous results indicate a connection of adult neurogenesis and NREM sleep. As WT animals showed a natural variance in the distribution of sleep stages, as well as, in the rate of adult hippocampal neurogenesis, we further examined whether the number of proliferating cells in the hippocampus correlated with the amount of NREM sleep.

**Figure 6:**
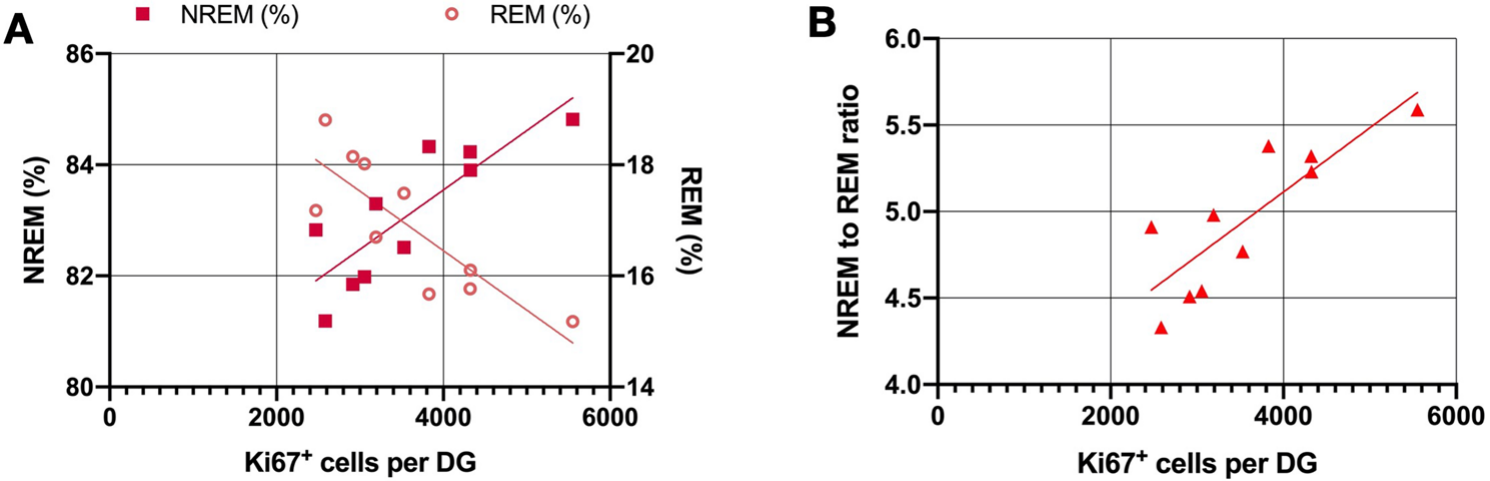
Correlation of Ki67^+^ cells and macroscopic sleep parameters during learning period in WT animals. (**A**) Correlation of Ki67^+^ cell number with NREM and REM sleep fraction. (**B**) Correlation of Ki67^+^ cell number with NREM to REM sleep ratio (r = 0.837; p = 0.003). (n = 10; Pearson’s correlation coefficient with two-sided test).

The number of Ki67^+^ labelled cells, indicating actively dividing precursor cells, strongly correlated with a shift towards a higher NREM sleep and, consecutively, smaller REM sleep fraction (higher NREM to REM sleep ratio) during the learning period (r = 0.837; p = 0.003; Fig. 6). Rate of adult hippocampal neurogenesis is associated with the NREM to REM sleep ratio as the main regulator of NREM sleep prolongation in post-learning sleep.

### Missing association of sleep and Morris water maze performance in Cyclin D2^−/−^ mice

**Figure 7:**
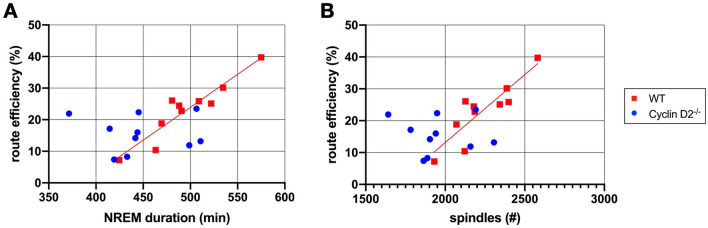
Correlation of different sleep parameters (mean d1-3) and route efficiency (mean d2-3). WT red, Cyclin D2^−/−^ blue. (**A**) Correlation of NREM sleep duration in min with route efficiency in % (WT: r = 0.936; p < 0.001; Cyclin D2^−/−^: r = − 0.036; p = 0.922). (**B**) Correlation of sleep spindles count with route efficiency in % (WT: r = 0.876; p = 0.001; Cyclin D2^−/^: r = − 0.071; p = 0.846). The regression line is only shown for the WT animals, as no association was found for the Cyclin D2^−/−^ mice. (Pearson’s correlation coefficient with two-sided test).

In the WT group, we found a strong positive correlation between mean NREM sleep duration per 24 h during the learning period and mean route efficiency at days 2 and 3 in the learning task (r = 0.936; p < 0.001; Fig. 7A), as well as between mean NREM sleep duration and search strategy (r = 0.686; p = 0.029; not shown). A similar correlation was found for the spindle count in the WT group (r = 0.876; p = 0.001; Fig. 7B). There were no associations between sleep parameters and MWM performance in the Cyclin D2^−/−^ mice lacking adult neurogenesis. No other correlations between sleep parameters and MWM performance were found.

Learning performance seemed to be independent of NREM sleep duration and spindle count in Cyclin D2^−/−^, whereas WT mice with longer NREM sleep duration and increased spindle count show higher route efficiency in the MWM.

## Discussion

In this study, we show that Cyclin D2^−/−^ mice that lack adult hippocampal neurogenesis show alterations of sleep micro- and macrostructure, which are associated with sleep-dependent memory consolidation. While others have shown that manipulating sleep affects adult neurogenesis (for an overview see Owen and Veasey, 2020^45^), this is the first study to demonstrate a potential reverse effect of adult neurogenesis on sleep. In order to link neurogenesis-related changes of sleep to memory consolidation, mice were confronted with a spatial memory task (MWM) for 3 consecutive days with 6 trials per day. We focused on measures that reflect hippocampal function and how they developed over several days after mice had had the opportunity to consolidate the new memory during sleep. Consistent with former studies, we found that Cyclin D2^−/−^ mice were not generally impaired in learning the spatial memory task, but rarely used the most efficient hippocampus-dependent search strategies and showed a lower route efficiency on day 2 and 3—after the first post-learning sleep. Route efficiency and search strategy depend on the use of a cognitive map encoded in the hippocampal formation^46,47^. The use of the most sophisticated search strategies has been shown to be a reliable qualitative parameter for hippocampus-dependent learning^39,47^. Accordingly, age-dependent decline of adult neurogenesis is associated with less frequent use of hippocampal search strategies^48^.

Recently, Petkova and colleagues provided evidence that Cyclin D2^−/−^ mice have substantial deficits in long-term memory formation. Interestingly, adult neurogenesis did not correlate with learning and short-term memory but with memory retention after 24 h^13^. This is in agreement with other reports demonstrating the role of adult hippocampal neurogenesis for long-term retention after days and weeks^49–51^. These observations underline the assumption that adult-born neurons are important for spatial memory consolidation which particularly happens during NREM sleep.

Learning is accompanied by changes in subsequent sleep macro- and microstructure supporting offline plasticity^3,25,26,52–54^. We hypothesized that adult neurogenesis, as a maximum variant of structural plasticity, plays a role in learning-related changes in sleep structure. Interestingly, we found a reduction of TST, NREM sleep duration and consequently NREM specific electrophysiological events, like SOs and spindles, in Cyclin D2^−/−^ mice during the baseline sleep recordings even in the absence of a robust learning stimulus. This finding may lead to the interpretation that Cyclin D2 depletion affects sleep regulation independently of impaired hippocampal neurogenesis and learning. However, the MWM just reflects an additional learning stimulus as even during baseline recording wake and behaving animals learn and display sleep-dependent memory consolidation. Therefore, differences in sleep under baseline conditions cannot be interpreted independently of hippocampal structure and function. In this context the MWM learning trials were used to stimulate memory-related sleep events (NREM-sleep, spindles, SO, PAC) and to correlate them with learning performance.

After exposing our mice to the MWM task, all animals showed an increased NREM sleep duration, which has been associated with the consolidation of spatial memory^55–57^. Although our study does not include an apparatus control of the learning task, we interpret the increase of NREM-sleep and spindle count as a consequence of the learning stimulus. Similar learning induced changes of sleep architecture had been demonstrated in detail previously^58–61^. The neuronal mechanisms that regulate memory consolidation and sleep are known to coordinate an optimal response to learning stimuli. For example, the macroscopic sleep structure of REM and NREM sleep may be differentially regulated depending on the type of stimulus during learning^62^. However, the neural mechanisms that are responsible for this regulation, as well as those which are targets, remain unknown. Interestingly, WT mice kept a constant TST by simultaneously reducing REM sleep and increasing NREM sleep. REM-sleep deprivation has been described to not have a negative effect in the MWM task^63^. Thus, increasing NREM sleep duration by shortening REM sleep duration constitutes an efficient way to improve spatial memory consolidation. In contrast, Cyclin D2^−/−^ mice increased NREM sleep mainly at the expense of longer sleep duration, keeping REM sleep duration similar to baseline. This is a less efficient way from an evolutionary perspective, where a longer TST might translate into a survival disadvantage.

As a consequence of the different strategies to prolong NREM sleep, NREM to REM sleep ratio, which was comparable at baseline, was higher in WT mice during the learning period as compared to Cyclin D2^−/−^ mice. From our data we suggest that adult hippocampal neurogenesis might play a regulatory role in this mechanism. The observation that the natural variance of cell proliferation in the adult hippocampus of WT animals highly correlated with the NREM to REM sleep ratio would confirm such interpretations. As we only measured cell proliferation at the end of the experiment but not the number of new-born and functional integrated neurons, it is also possible that changes in cell proliferation is a consequence of increased NREM-ratio rather than the cause. However, this explanation does not exclude the assumed impact of hippocampal plasticity (new-born neurons) on sleep architecture, and could rather indicate that sleep may function as regulative event that adjusts the rate of neurogenesis according to the exposure to new memories.

At the microstructural level we focused on electrophysiological events that are known to facilitate sleep-dependent memory consolidation like sleep spindles and SO. During learning days both groups showed an increase in spindle number compared to baseline sleep, but only WT animals displayed and increased spindle density.

Correlational analyses of sleep parameters during the learning days and water maze performance revealed that in WT animals a higher NREM sleep duration and more sleep spindles were associated with better task performance, while this relationship was absent in Cyclin D2^−/−^ mice. We did not find further associations between sleep parameters and MWM performance, which might be due to the lack of an appropriate recall test (probe trial). As we cannot draw causal conclusions with our experimental setup, it is of course possible that the relationship could be bidirectional, as higher route efficiency may also lead to longer NREM sleep duration and more sleep spindles.

Sleep spindles are a hallmark of sleep-dependent memory consolidation, and they increase as a reaction to learning stimuli^25,26,52–54^. They also positively correlate with certain aspects of memory and learning performance^25,26,64^. Thalamic sleep spindles occur in synchrony with hippocampal SWRs to facilitate communication between the hippocampus and neocortex during sleep-dependent memory consolidation process^24,65,66^. In contrast to WT mice, Cyclin D2-/- mice did not show a learning-induced increase of spindle density. We therefore assume that attenuation of adult neurogenesis impairs the interaction between the hippocampus and neocortex during sleep. There have been speculations about how adult hippocampal neurogenesis might be linked to these processes^67–69^. Recently, Berdugo-Vega et al. showed that artificially increasing adult hippocampal neurogenesis led to changes in CA3-generated SWRs^70^. With our recording setup we could not measure hippocampal SWRs directly. However, as spindles and ripples occur in time-locked spindle-ripple events^3^, differences in spindle count and density between WT and Cyclin D2^−/−^ animals are likely linked to SWR changes that are regulated by the rate of neurogenesis.

Sleep spindles are also known to have a stabilizing effect on NREM sleep in humans and rodents and elevate the arousal threshold^44,71^. In line with these observations we found a higher arousal index and reduced NREM to REM sleep ratio in mice with less spindles. Cyclin D2^−/−^ mice did not show any changes in spindle density during learning days in contrast to WT animals. Whether adult neurogenesis affects the stabilization of NREM sleep via the promotion of hippocampal SWRs and spindles has to be confirmed in future experiments.

Furthermore, PAC has been shown to be an important aspect of sleep-dependent memory consolidation^41,43^. We found the coupling between delta and spindle band during NREM sleep to be reduced in the Cyclin D2^−/−^ group. This result indicates an impaired coordination of SO and sleep spindles in mice lacking adult hippocampal neurogenesis as a signature of memory impairment. A similar reduction in the coupling of hippocampal and cortical activity patterns during sleep has been reported by Zhurakovskaya et al., 2019 in APP/PS1 mice—a widely used Alzheimer’s disease model^72^. Moreover, it is known that adult hippocampal neurogenesis is reduced in Alzheimer’s disease in humans^73^. An impaired synchrony between SOs and spindles during sleep as a consequence of aging has also been described^74^. At the same time, aging is probably the most robust natural negative regulator of baseline adult neurogenesis. Thus, the loss of cellular plasticity in the hippocampus by aging or neurodegenerative diseases might be involved in impaired sleep-related network interactions.

Although, we cannot draw clear causal conclusion from our data on how Cyclin D2 depletion affects the interaction of adult neurogenesis, sleep and memory formation, we suggest an interdependent relationship of these events with adult neurogenesis being the superordinate modulator of memory and memory-related sleep changes. It is well established that Cyclin D2 is essential for neuronal precursor cell proliferation in the adult brain. It is therefore unlikely that impaired adult neurogenesis is the result of potentially Cyclin D2^−/–^induced NREM sleep alterations, but rather the cause for spatial memory deficits as well as disruption of quantitative and qualitative sleep parameters, which are known to be associated with memory formation.

The depletion of Cyclin D2 not only affects adult neurogenesis but also several other physiological functions, including B cell proliferation^75^ and differentiation of cerebellar granule cells and stellate interneurons^76^. Therefore, the effects found might not exclusively be attributed to the lack of adult-born neurons. However, at the behavioral level most phenotypic observations are related to hippocampal function, including impaired nest construction, digging, marble burying^77^, and spatial memory formation like the MWM^31^. Thus, it has been suggested that these behavioral deficits result from deprived adult neurogenesis and smaller hippocampal volume respectively. We here demonstrate a new aspect of the Cyclin D2^−/−^ phenotype related to changes in sleep macro- and microstructure. Similar changes have been observed in other models of hippocampal damage or dysfunction. Several clinical sleep studies including patients with hippocampal pathology also indicate a substantial role of the hippocampus in sleep physiology. Bilateral hippocampal damage results in a virtual loss of NREM slow wave sleep compared to healthy controls^22^. Lucey et al., 2019 showed that NREM slow wave activity is reduced in early Alzheimer’s disease and inversely correlates with tau pathology^21^. In line with these observations we suggest, that changes in sleep micro- and macrostructure in Cyclin D2^−/−^ mice most likely result from structural changes within the hippocampus.

Taking all findings into account, we suggest an important role of neurogenesis in sleep-dependent memory consolidation processes and memory-associated sleep regulation.

Very recently Kumar et al. published an outstanding and comprehensive study demonstrating that adult-born hippocampal granule cells are reactivated during REM sleep after contextual fear learning. Optogenetic silencing of these adult-born neurons during REM sleep impaired memory consolidation^19^. NREM and REM sleep seem to cover different aspects of memory formation. REM sleep theta activity has been brought into connection with emotional and procedural memories, while NREM sleep SOs, spindles and SWRs mediate the consolidation of declarative memories^3,15,78^. In contrast to this dual process hypothesis, some authors support the idea that NREM and REM sleep might interact complementary to promote the same types of memory^65,79^. Natural sleep architecture is characterized by an alternating sequence of NREM and REM sleep. NREM sleep always precedes REM sleep. Within the context of memory formation and plasticity it has been postulated that REM sleep serves as a function that complements that of prior NREM sleep^80,81^. Kumar and colleagues found a sparse firing rate of 4-week-old adult-born hippocampal neurons during REM sleep, which was reduced after acquisition of contextual fear memory. However, silencing or random activation of adult-born neurons impaired memory formation^19^. Thus, the sparse activity seems to be necessary for REM sleep dependent memory consolidation. In line with the sequential hypothesis of REM and NREM sleep function, NREM oscillations (spindles and ripples) have been shown to be a strong predictor of REM-related decrease in hippocampal firing rate^80^. In the context of this NREM-REM interplay our interpretation of the role of adult neurogenesis for NREM sleep-related memory consolidation may perfectly complement the finding of Kumar et al. Whether the activity of adult-born granule cells during NREM sleep influences their REM-related activity afterwards and thereby supports the processing of hippocampal memories across the sleep cycle would be an interesting question for further experiments.

Sleep at the behavioural level and adult neurogenesis at the structural level are both essential for long term memory formation. Our data support the idea that these two events are interdependent in terms of function and regulation. From a medical perspective, the understanding about the strong relationship of neuronal plasticity and sleep is important for preventive approaches, as sleep disturbances become more frequent during aging, increase the risk for cognitive decline and also represent a common symptom even in early stages of neurodegenerative diseases.

## Supplementary information

Supplementary information.

## Data Availability

The datasets generated during and/or analysed during the current study are available in the EBRAINS repository https://kg.ebrains.eu/search/instances/Dataset/830f33f1-cb6e-45ac-9203-11f23e97c273.
