# Supplementary material for: Disruption of NREM sleep and sleep-related spatial memory consolidation in mice lacking adult hippocampal neurogenesis
Source: Sci Rep. 2020 Oct 5;10:16467. doi: 10.1038/s41598-020-72362-3 (PMC7536189; doi:10.1038/s41598-020-72362-3)
Supplement: Supplementary file 1 — Supplementary information. [file 41598_2020_72362_MOESM1_ESM.docx]

Supplementary information

Disruption of NREM sleep and sleep-related spatial memory consolidation in mice lacking adult hippocampal neurogenesis

D. Sippel^1,2^, J. Schwabedal^3^, J. C. Snyder^4^, C. N. Oyanedel^1^, S. N. Bernas^6^, A. Garthe^5^, A. Tröndle^4,6^, A. Storch^7,8^, G. Kempermann^5,6^, M. D. Brandt^4,5,^*

^1^ Institute of Medical Psychology and Behavioral Neurobiology, University of Tübingen, 72076 Tübingen, Germany

^2^ Department of Psychiatry and Psychotherapy, University Hospital Tübingen, 72076 Tübingen, Germany

^3^ Max Planck Institute for the Physics of Complex Systems, 01187 Dresden, Germany

^4^ Department of Neurology, University Hospital, Technische Universität Dresden, 01307 Dresden, Germany

^5^ German Center for Neurodegenerative Diseases (DZNE) Dresden, 01307 Dresden, Germany

^6^ Center for Regenerative Therapies TU Dresden, 01307 Dresden, Germany

^7^ German Center for Neurodegenerative Diseases (DZNE) Rostock, 18147 Rostock, Germany

^8^ Department of Neurology, University of Rostock, 18147 Rostock, Germany

* Corresponding Author

*
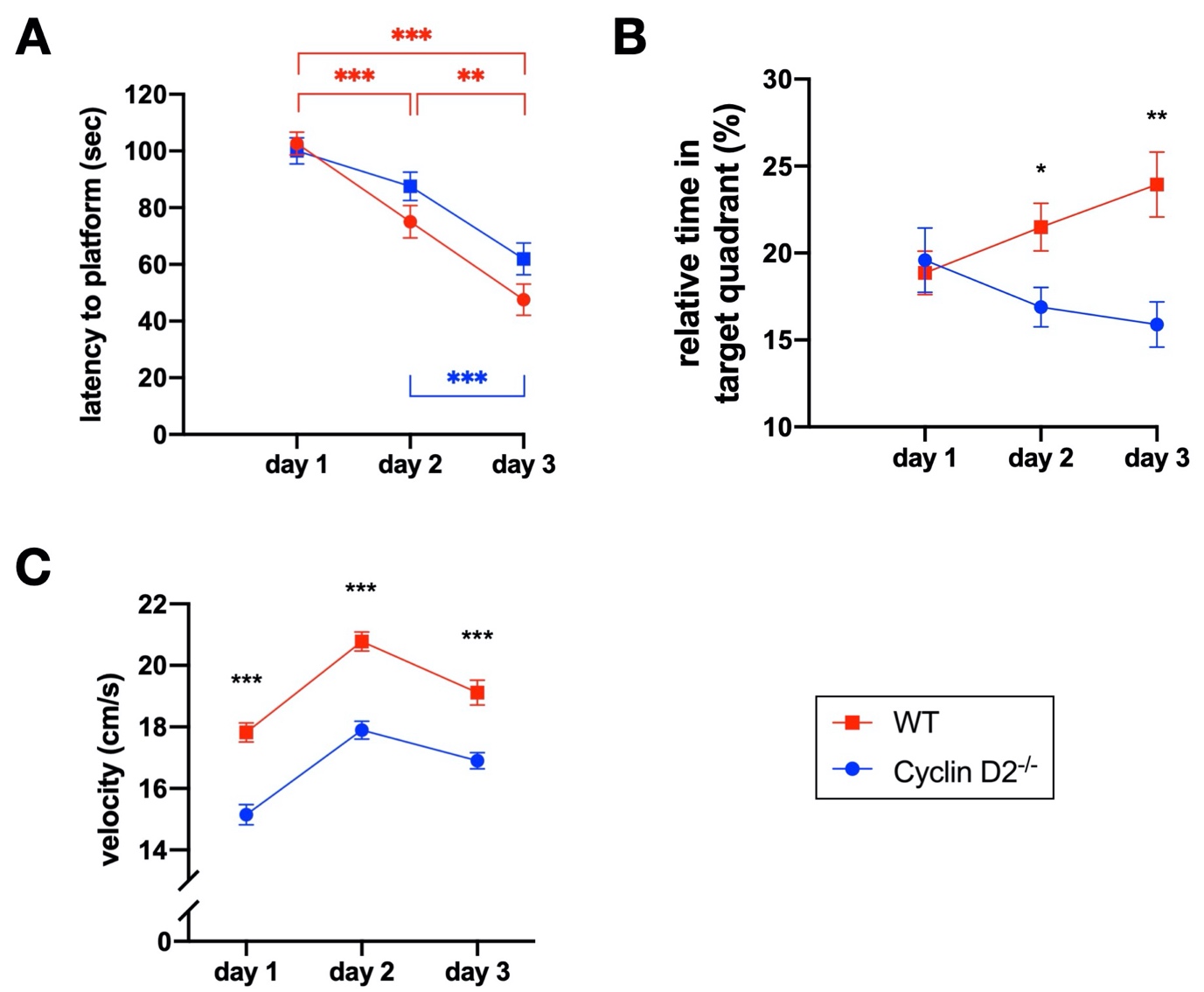
*

***Supplementary Figure 1****: Additional MWM readouts as mean values for each day.* ***A.*** *Both, WT (red) and* Cyclin D2^-/-^ (blue) showed a reduction in latency to find the platform during the 3-day learning period, whereas only WT animals significantly shorten latency between day 1 and 2. **B.** WT animals spend more time in the target quadrant during learning trails at day 2 and 3 compared to Cyclin D2^-/-^ *mice. Please note that no probe trial (removed platform) was performed. Hence, time in target quadrant is influenced by search strategy as well as latency to find the platform.* ***C.*** *Swimming speed slightly but significantly differ between WT and* Cyclin D2^-/-^. As differences in velocity, which is unrelated to memory, might have an impact on timed-dependent measurements we focused on readouts that more precisely represents hippocampal function (route efficiency and strategy, Fig. 2). *(n = 10 per group, one-way repeated-measure ANOVA with post-hoc Bonferroni-adjusted two-sided t-test as appropriate *: p < 0.05; **: p < 0.01; ***: p < 0.001; data are presented as mean ± 1 SEM)*


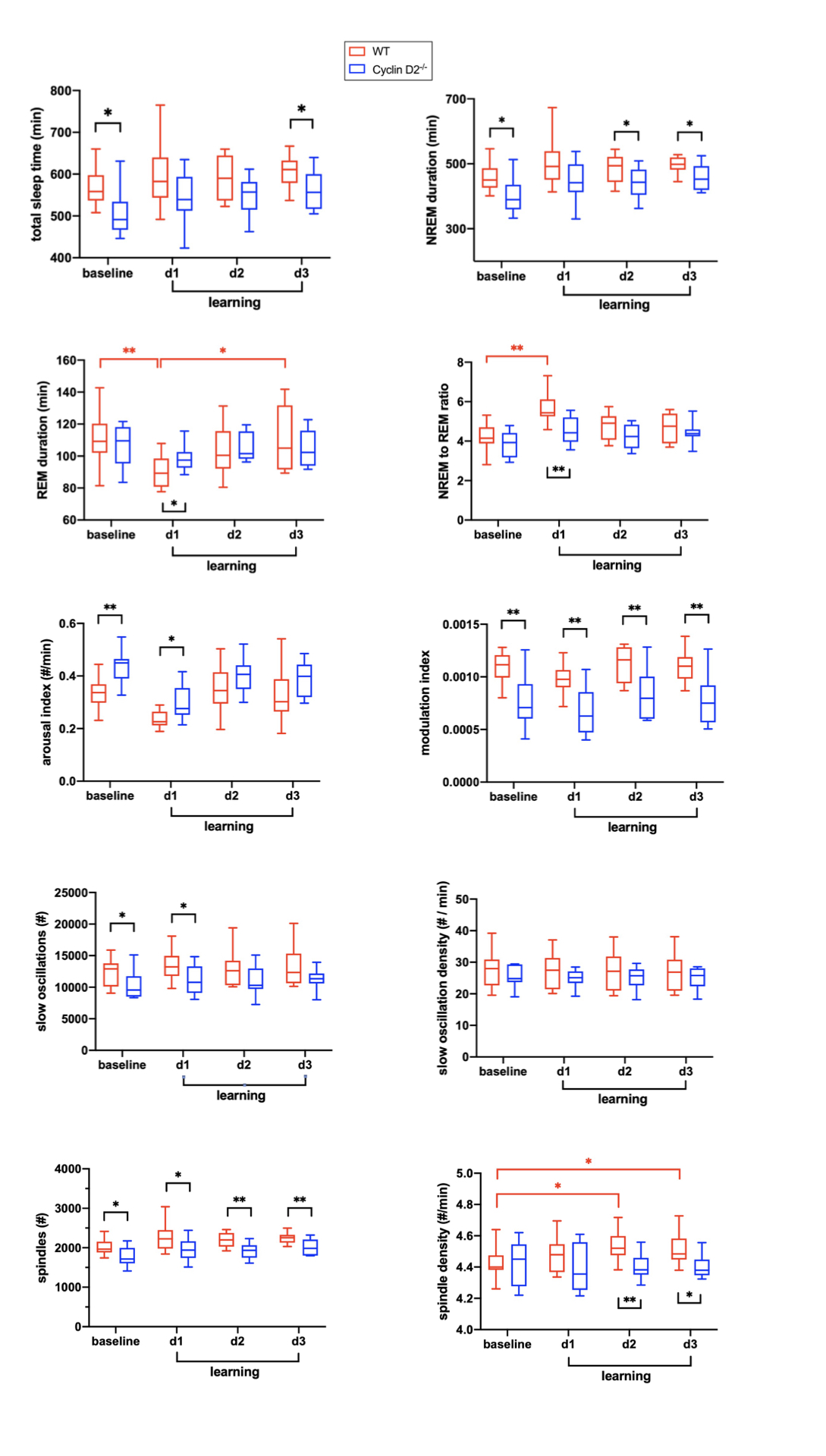


***Supplementary Figure 2****: Sleep parameters for each day of the experiment. The finer resolution of sleep parameters revealed a prompt change of sleep macrostructure (NREM-REM ratio) after the first learning day and a delayed increase of sleep microstructure (spindles) at day 2 and 3. (n = 10 per group; two-sided unpaired t-test for group comparison and one-way repeated-measure ANOVA with post-hoc Bonferroni-adjusted two-sided t-test as appropriate; *: p < 0.05; **: p < 0.01).*

*For the main analysis of learning-induced sleep changes, sleep data from days 1, 2, and 3 of the MWM were averaged (see results, Fig. 4 and 5). This was done for several reasons: I) It has been previously described that there are at least two types of consolidation, a fast (synaptic level) and a slow (systems consolidation) process. The latter takes place over several days (Dudai, 2004; Frankland and Bontempi, 2005). II) It is comprehensible that there is a bidirectional link: learning stimuli per se may influence sleep-structure and sleep-dependent neuronal activity (Peters et al., 2007; Hellman and Abel, 2007; Gais et al., 2002), while sleep quality influences learning performance (Tartar et al., 2006; McCoy and Strecker, 2011). III) As we did not include a probe trial to test recall performance every day, our paradigm represents a continuous learning task over 3 days without having the option to compare sleep and learning performance at single days.*

*Literature:*

- *Dudai, Y. The neurobiology of consolidations, or, how stable is the engram? Annual review of psychology 55, 51–86 (2004). DOI: 10.1146/annurev.psych.55.090902.142050*
- *Frankland, P. W. & Bontempi, B. The organization of recent and remote memories. Nature reviews. Neuroscience 6, 119–130 (2005). DOI: 10.1038/nrn1607*
- *Peters, K. R., Smith, V. & Smith, C. T. Changes in sleep architecture following motor learning depend on initial skill level. J Cogn Neurosci 19, 817-829 (2007). DOI: 10.1162/jocn.2007.19.5.817*
- *Hellman, K. & Abel, T. Fear conditioning increases NREM sleep. Behav Neurosci 121, 310-323 (2007). DOI: 10.1037/0735-7044.121.2.310*
- *Gais, S., Molle, M., Helms, K. & Born, J. Learning-dependent increases in sleep spindle density. J Neurosci 22, 6830-6834 (2002). DOI: 10.1523/JNEUROSCI.22-15-06830.2002*
- *Tartar, J. L. et al. Hippocampal synaptic plasticity and spatial learning are impaired in a rat model of sleep fragmentation. Eur J Neurosci 23, 2739-2748 (2006). DOI: 10.1111/j.1460-9568.2006.04808.x*
- *McCoy, J. G. & Strecker, R. E. The cognitive cost of sleep loss. Neurobiol Learn Mem 96, 564-582 (2011). DOI: 10.1016/j.nlm.2011.07.004*
